# Home Treatment and Perinatal Psychiatry: An Alternative to Acute Psychiatric Wards

**DOI:** 10.1192/j.eurpsy.2024.1684

**Published:** 2024-08-27

**Authors:** J. Marti Bonany, D. García Hernández, R. Romar Navia, D. Tolosa Merlos, F. Casanovas Martínez, C. Llimona Sánchez, A. Pérez Oms, G. De Iturbe Catania

**Affiliations:** ^1^Salut Mental Institut, Hospital del Mar, Barcelona; ^2^Hospital Universitari Son Espases, Mallorca, Spain

## Abstract

**Introduction:**

Women experiencing severe perinatal mental health problems require specialized services and care. Perinatal mental disorders are common and can contribute to maternal mortality, affecting neonatal, infant, and child outcomes. Home treatment can prevent hospital admissions and promote strategies within the patient’s support network.

**Objectives:**

Our aim is to describe a clinical case in perinatal psychiatry managed by a Psychiatric Home Treatment Unit.

**Methods:**

We present a case of perinatal psychotic depression in a 26-year-old pregnant woman.

**Results:**

We describe the case of a patient with no prior history of mental health issues. She was 25 weeks pregnant when she first sought psychiatric help in July 2023 and was diagnosed with depressive disorder with psychotic symptoms. She reported symptoms such as low mood, psychomotor inhibition, delusional guilt thoughts, and auditory hallucinations beginning three weeks before her initial visit. Due to her clinical presentation, the patient was admitted to the hospital, where pharmacological treatment was initiated with Olanzapine 5 mg, Sertraline 50 mg, and Lorazepam 1.5 mg. She remained in the hospital for four days, during which she showed gradual improvement but did not achieve full recovery.

Considering the improvement observed, home treatment was proposed and accepted by the patient and her relatives. During home treatment, she continued to exhibit persistent depressive and psychotic symptoms, including low mood, inhibition, and delusional thoughts of ruin and catastrophe. Therefore, her treatment was adjusted, with Olanzapine increased to 10 mg, Sertraline raised to 100 mg, and Lorazepam reduced to 0.75 mg. Over time, significant improvement in her clinical symptoms was noted. Throughout the follow-up period, she reported no significant side effects from the pharmacological treatment. After a month of follow-up in our department, she was discharged with outpatient care provided by a specialized community perinatal psychiatric unit.

**Conclusions:**

We illustrate the possibility of home treatment for perinatal psychiatric disorders. The potential benefits of remaining close to one’s support network and developing coping strategies can be advantageous during the course of illness. Further studies should be conducted to explore these potential benefits.

**Disclosure of Interest:**

None Declared

